# Kerf-Less Exfoliated Thin Silicon Wafer Prepared by Nickel Electrodeposition for Solar Cells

**DOI:** 10.3389/fchem.2018.00600

**Published:** 2019-01-14

**Authors:** Hyun-Seock Yang, Jiwon Kim, Seil Kim, Nu Si A. Eom, Sangmuk Kang, Chang-Soon Han, Sung Hae Kim, Donggun Lim, Jung-Ho Lee, Sung Heum Park, Jin Woo Choi, Chang-Lyoul Lee, Bongyoung Yoo, Jae-Hong Lim

**Affiliations:** ^1^Electrochemistry Department, Korea Institute of Materials Science, Changwon, South Korea; ^2^Department of Physics, Pukyong National University Busan, South Korea; ^3^Department of IT Convergence, Korea National University of Transportation, Chungju, South Korea; ^4^Laser Advanced System Industrialization Center, Mam-myeun, South Korea; ^5^Department of Materials Engineering, Hanyang University, Ansan, South Korea; ^6^Advanced Photonics Research Institute, Gwangju, South Korea

**Keywords:** ultra-thin silicon wafer, spalling, stressor layer, kerf loss, edge slope, electrodeposition

## Abstract

Ultra-thin and large-area silicon wafers with a thickness in the range of 20–70 μm, were produced by spalling using a nickel stressor layer. A new equation for predicting the thickness of the spalled silicon was derived from the Suo–Hutchinson mechanical model and the kinking mechanism. To confirm the reliability of the new equation, the proportional factor of stress induced by the nickel on the silicon wafer, was calculated. The calculated proportional factor of λ = 0.99 indicates that the thickness of the spalled silicon wafer is proportional to that of the nickel layer. A similar relationship was observed in the experimental data obtained in this study. In addition, the thickness of the stressor layer was converted to a value of stress as a guide when using other deposition conditions and materials. A silicon wafer with a predicted thickness of 50 μm was exfoliated for further analysis. In order to spall a large-area (150 × 150 mm^2^ or 6 × 6 in^2^) silicon wafer without kerf loss, initial cracks were formed by a laser pretreatment at a proper depth (50 μm) inside the exfoliated silicon wafer, which reduced the area of edge slope (kerf loss) from 33 to 3 mm^2^. The variations in thickness of the spalled wafer remained under 4%. Moreover, we checked the probability of degradation of the spalled wafers by using them to fabricate solar cells; the efficiency and ideality factor of the spalled silicon wafers were found to be 14.23%and 1.35, respectively.

## Introduction

Silicon solar cells are the focus of considerable research efforts because of their high energy-conversion efficiency (~25%) (Green et al., 2015), stability, and so on (Bruel, 1995; Dross, 2008; Shahrjerdi et al., 2012; Radhakrishnan et al., 2014; Kobayashi et al., 2015; Lee et al., 2016; Green et al., 2017; Wang et al., 2017). Ultra-thin silicon wafers with thickness in the range of 40–60 μm are particularly suitable for high-efficiency solar cells because of their high light absorption and flexibility (Dross, 2008). However, the >100% kerf loss during the fabrication of ultra-thin silicon wafers (thickness: <100 μm) using conventional sawing technology is a critical problem (Green et al., 2017) that increases material cost and requires additional post-sawing processes. Therefore, it is important to minimize the waste associated with wafer losses during sawing. Several methods are available for fabricating kerf-less thin silicon wafers, such as the stress-inducing process (Shahrjerdi et al., 2012; Wang et al., 2017), ion implantation (Bruel, 1995; Lee et al., 2016), and epitaxial growth (Radhakrishnan et al., 2014; Kobayashi et al., 2015). Since it is difficult to form a stable trajectory during the ion-implantation process, the resulting wafers tend to have high surface roughness (Suo, 1989). The process of inducing stress through by mismatching the thermal coefficients of silicon and a deposited polymer requires a high temperature, which causes degradation of the carrier lifetime (Suo and Hutchinson, 1989). Slim-cut and epitaxial-growth processes require complex equipment and procedures (Radhakrishnan et al., 2014). On the other hand, the electrochemical process is quite suitable because of the low production cost, easy scale-up, stress control, and high material yield (Drory et al., 1988). However, it is difficult to predict the propagation and initial depth inside a silicon wafer, creating problems in the thickness control of spalled silicon wafers, which represent a major disadvantage of the electrochemical process when compared with other methods. In addition, the general electrochemical process causes edge sloping up through the threshold of steady-state crack depth during spalling (Suo, 1989), which causes problems such as kerf loss at the edge of the wafer and high roughness which can cause fractures in the spalled silicon wafer. The fractures induced by kerf loss can be a critical problem for the large-scale production of spalled silicon wafers with areas over 150 × 150 mm^2^ (6 × 6 in^2^). It is therefore necessary to reduce the edge slope and predict the thickness of the spalled silicon wafer.

In the study reported here, we combined the process of laser pretreatment at the edge of a silicon wafer and electrodeposition of nickel, with high internal stress, on top of the substrate. This structure was designed to reduce the edge slope and enhance the uniformity of the silicon wafer after spalling. In addition, a relationship was proposed for predicting the thickness of the spalled silicon wafer. The long-wavelength laser process formed cracks without damaging the surface of the sample, which served as the initial cracks that extended along the interface, deviated into the substrate, and subsequently propagated in a direction parallel to the interface at a steady-state crack depth beneath the interface (Suo and Hutchinson, 1990). We confirmed the effect of the initial crack formed by the laser process on the kerf-less silicon wafer. A new equation for predicting the spalling silicon thickness was proposed, based on the prediction of the initial crack depth and the calculation of the steady-state crack depth from the Suo–Hutchinson (S&H) model (Suo and Hutchinson, 1990). The validity of the proposed equation was evaluated by comparing the calculated results with the experimental data.

## Experimental

**Figure 1:**
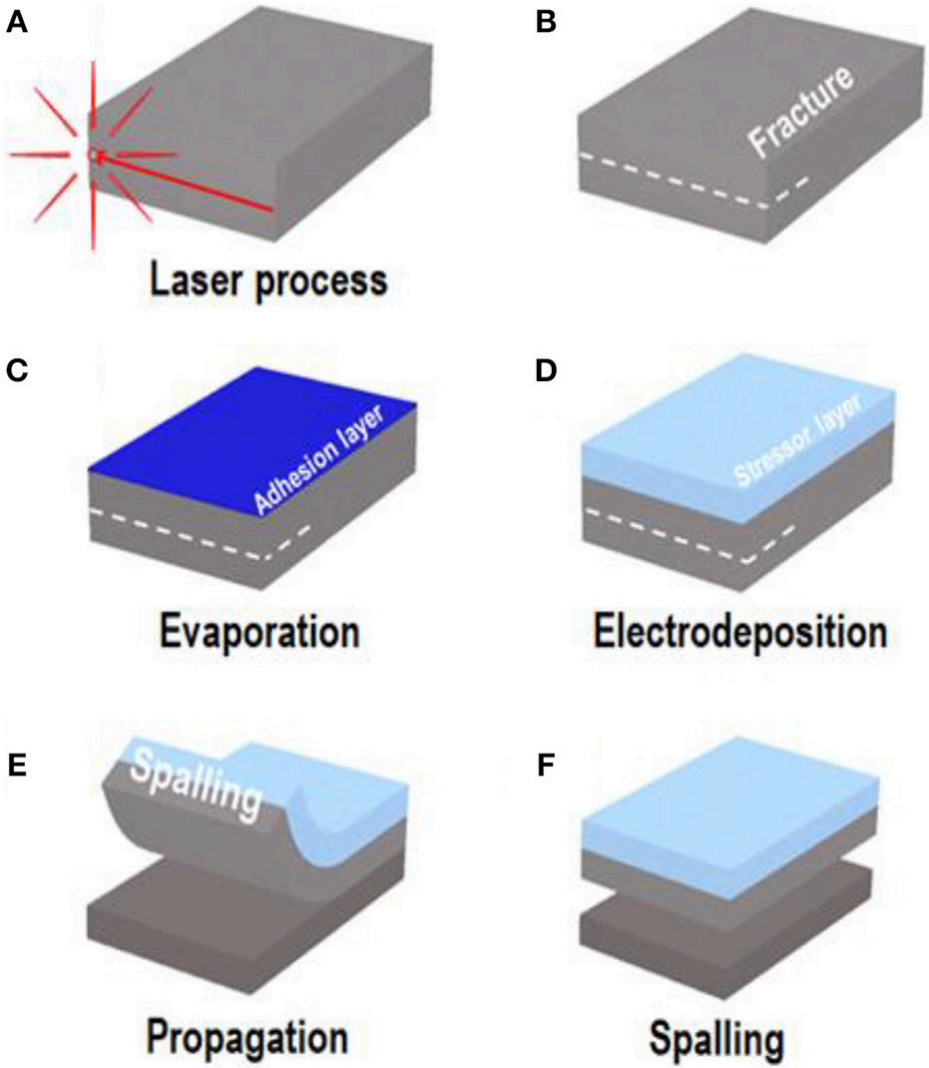
Schematic of the laser-based spalling process: **(A)** laser process at the edge of silicon wafer; **(B)** formation of a fracture at the wafer edge; **(C)** e-beam evaporation for adhesion layer and seed layer; **(D)** electrodeposition to grow stressor layer; **(E)** spontaneous crack and propagation of the tip; **(F)** spalled kerf-less silicon wafer.

The spalling process can be divided into three successive steps: (1) pretreatment with a laser to form cracks at the edge of the wafer; (2) electrodeposition of the metal stressor layer; and (3) spalling of the silicon wafer. A schematic diagram of the process is shown in Figure 1.

In our study, p-type monocrystalline 150 × 150 mm^2^ (6 × 6 in^2^) silicon wafers with <100> orientation and 1–10 *Ω* resistance were utilized because their low roughness was suitable for crack propagation. In order to reduce the edge slope after spalling, pretreatment was carried out using a laser (Lumera Hyper Rapid 50, Coherent, USA). The laser wavelength was set at 1,064, 532 or 355 nm, the generation capacity was selectable with a power of 50, 20, or 16 W, and the frequency was 400 KHz. The laser was focused at a point in an area that had the same steady-state crack depth, to form initial cracks all around the edge of the silicon wafer at a periodic distance of 100 μm. After the laser treatment, an electron-beam (e-beam) evaporator (Super High Speed Evaporator System, Daedong Hightec, Korea) was used to deposit Ti as an adhesion layer (thickness: 20 nm) and nickel as a seed layer (thickness: 100 nm) on the silicon wafer. The nickel seed layer had much higher conductivity than the silicon wafer. Prior to electrodeposition, the wafer was degreased in an alkaline bath (5% NaOH) to increase the hydrophilicity of its surface, followed by pickling in a 10% HCl bath to remove any metal oxide.

After the wafer was cleaned and treated, nickel(II) chloride (NiCl_2_; concentration: 1 mol/L, purity: 98.5%, SAMCHUN, Korea) and sodium citrate (concentration: 0.1 mol/L, purity: 99%, Sigma Aldrich, USA) were mixed together to form the electrodeposition bath; a sufficient amount of HCl was added to adjust the pH of the mixture to 3.5. NiCl_2_ was the main supplier of nickel ions, and sodium citrate served as a buffer to maintain the pH and carry the electrons in the bath. The nickel stressor layer was deposited on the silicon wafer by immersing it in the all-chloride bath. This was done because a higher internal stress could be obtained than in an all-chloride bath, than in a non-chloride bath (Bedell et al., 2017). A low voltage (1.2–2.8 V) was applied by a power supply with a direct current, and a nickel stressor layer with a thickness of 50 μm was obtained after 250 min. The current density used for the nickel electrodeposition was 5 mA/cm^2^, and the bath temperature was maintained at 50. The thickness and variations in thickness of the deposited nickel stressor layer, were measured by analyzing scanning electron microscope (SEM; SU-6000, Hitachi, Japan) cross-sectional images and using an X-ray fluorescence thickness analyzer (D/MAX-2500, Rigaku, Japan). In addition, the elemental detection and crystal structure of the spalled silicon wafer were measured by secondary ion mass spectroscopy (SIMS; IMS 7f, CAMECA, France) and a X-ray diffraction (XRD; D/Max-2500VL, Rigaku, Japan), *respectively*. Steady-state photoluminescence (PL) spectra were measured using a monochromator (Acton Series SP-2150i, Princeton Instruments, USA) equipped with a photomultiplier tube (PMT; ID-441 for Acton Series, Princeton Instruments, USA) and a Ti:sapphire excitation laser with a wavelength of 860 nm (Mira 900, Coherent, USA).

A wafer sample, which did not have spontaneous cracks formed during nickel electrodeposition, was removed from the electrodeposition bath. If there was sufficient breaking stress within the silicon wafer, the fracture formed by laser treatment would have propagated through the sample itself. After spalling, the nickel layer was etched by a mixed solution consisting of nitric acid (mixing ratio of 1:1), deionized water, and 25 mL/L HF. The etching of the Ni layer and Ti adhesion layer and the diffusion of the impurities into the silicon wafer were measured by SIMS.

The stress induced in the nickel layer was measured by a stress strip test (B975, Specialty Testing, USA). The stress depended on the grain size of the nickel particles, deposition rate, and potential difference. Moreover, the most critical factor of stress control was the thickness of the nickel layer. The edge slope of the spalled silicon wafer was measured with an optical microscope (RH-2000, Hirox, Japan) and by thickness profiling (RH-2000, Hirox, Japan).

Prior to the fabrication of the solar cell, the spalled silicon wafer was sequentially cleaned as follows: (1) a short dip in 5 M hydrofluoric (HF) acid; (2) immersion in 50 wt% potassium hydroxide (KOH) at a temperature of 80°C for 1 min; (3) immersion in a piranha solution (H_2_SO_4_/H_2_O_2_ volume ratio = 3:1) for 15 min; and (4) a short dip in HF to remove unwanted contaminants from the spalling process. To increase light absorption, pyramid textures were formed on the front side of the wafer by treating it with 700 mL of a 2 wt% KOH solution mixed with 45 mL of isopropyl alcohol (IPA) at a temperature of 80°C for 1 h.

A silicon solar cell with a dopant-free heterojunction was fabricated using the cleaned and textured spalled silicon wafer. First, an ultra-thin Al_2_O_3_ layer (thickness: 0.5 nm) was deposited using an atomic layer deposition (ALD) system (D100, NCD Tech, Korea) at a temperature of 150°C to lightly passivate the surface. Next, a layer of lithium fluoride (LiF_*x*_; purity: 99.98%, LTS chemical, USA) (thickness: 1 nm) was deposited by evaporation as an electron-transporting layer and an Al layer (thickness: 100 nm) was deposited without a vacuum break on the rear side of the wafer. A vanadium oxide (V_2_O_*x*_; purity: 99.99%, LTS Chemical, USA) layer (thickness: 15 nm) was deposited in the same manner as a hole-transporting layer on the front side of the wafer. Subsequently, a layer of indium–tin oxide (ITO; 10% SnO_2_, 90% In_2_O_3_, purity: 99.99%) (thickness: 80 nm) was sputtered onto the V_2_O_*x*_ surface in an Ar flow at room temperature under a pressure of 8.0 × 10^−7^ Torr for 1070 s. Finally, an Ag layer (thickness: 1 μm) was deposited by evaporation as the electrode on the front side.

The current density vs. voltage (*J*–*V*) data of the solar-cell performance was obtained with a solar simulator (XES-502S, San-El Electric, Japan) under one sun irradiation (100 mW/cm^2^, AM 1.5 spectrum, 25°C). The ideality factor (η) and series resistance (*R*_S_) were extracted from the dark *J*–*V* curve. The spectral response measurements were obtained as the incident-photon-to-current conversion efficiency (IPCE; k3100, PV Measurements, USA) using a 150 W arc lamp with a wavelength range of 350–1,100 nm.

## Results and Discussions

### Stress Induced Stress by Nickel Stressor Layer

**Figure 2:**
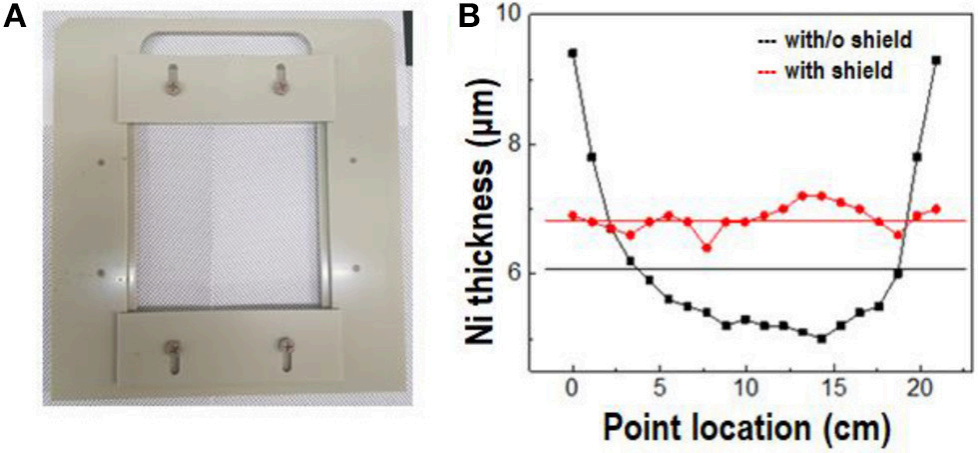
**(A)** Image of shield for uniform deposition of the nickel stressor layer. **(B)** Variations in thickness of nickel stressor layers, deposited without the shield (black line), and with the shield (red line).

For delivery of uniform stress on a large silicon wafer, the nickel stressor layer must be uniformly deposited. Since the trajectory of spalling propagation was parallel to the nickel surface, the control of uniform deposition of the nickel stressor layer was a critical factor of large-area spalling (Suo and Hutchinson, 1990). Figure 2 shows a shield, with dimensions of 12.48 × 12.48 cm^2^, used for the nickel deposition. It was placed 2 cm from the substrate to reduce the thickness variation (Drory et al., 1988). Figure 2B shows the thickness of the nickel layer at different points in the diagonal direction. Without a shield, the thickness of the deposited nickel film at the edge and center, and the mean thickness were approximately 9.5, 5, and 6 μm, respectively. On the other hand, the nickel film deposited with a shield had a uniform thickness of about 7 μm, which demonstrates that the shield assisted uniform growth of the nickel stressor layer, when it was deposited by controlling the electric field in the bath.

**Figure 3:**
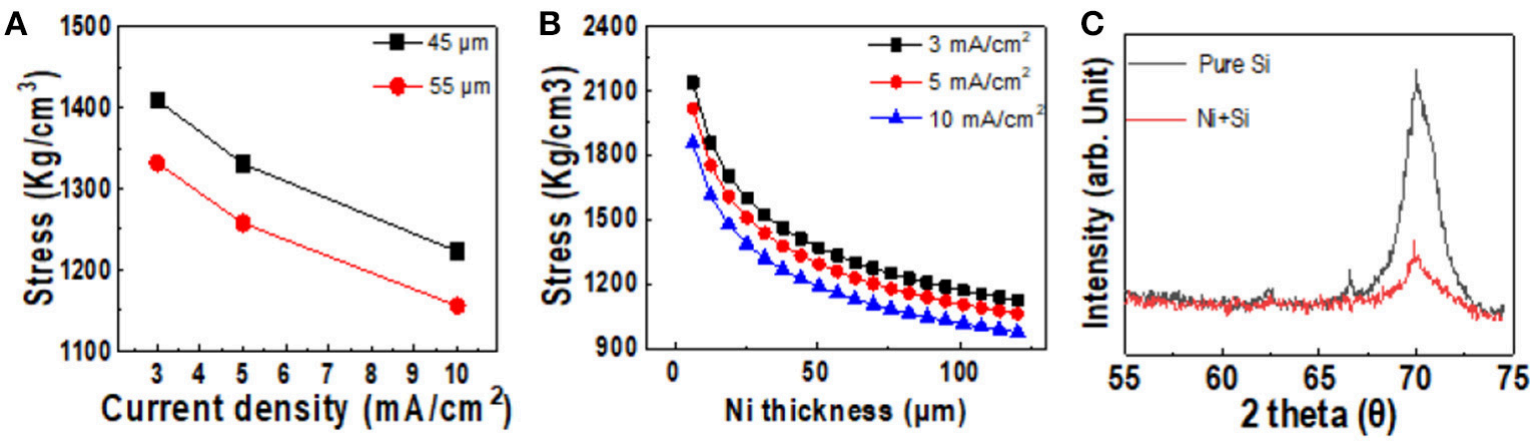
Stress induced by the nickel stressor layer as a function of the **(A)** current density and **(B)** thickness of nickel layer. **(C)** Shift in XRD peak caused by the stressor layer.

Figure 3 shows the stress of different nickel layers formed under various deposition conditions. Tensile stress was induced on the silicon wafer because the nickel layer had a smaller lattice constant (3.520 Å) than the silicon wafer (5.430 Å for Si <100>) (Bilby and Eschelby, 1968). Figure 3A shows the intrinsic stress of the deposited nickel stressor layers, with a thickness of 45 and 55 μm, as a function of the current density (3, 5, and 10 mA/cm^2^). The intrinsic stress decreased with increasing current density, indicating that higher intrinsic stress was induced in the thinner silicon substrate. Although all the stressor layers had the same thickness, the amount of induced stress changed with the current density, which meant that an optimized current density was necessary (Durney, 1984). To optimize the current density, the stress induced by the intrinsic stress in the nickel stressor layer, was measured by a stress strip test; the results are shown in Figure 3B. The stress induced by the nickel layer decreased with increasing nickel-layer thickness, because the stress was transferred from the nickel layer to the silicon wafer. When a high level of intrinsic stress (1,260 mA/cm^2^) was induced, a spontaneous crack emerged at the edge of the sample and caused high roughness on the spalled silicon wafer. On the other hand, when a lower level of induced stress (1,600 mA/cm^2^) was not enough to cause spalling, the initial crack at the edge of the silicon wafer did not cause any breakage (Supplementary Figure 1). If the external force for spalling was applied to the silicon wafer's edge, the nickel layer would be torn because of low induced stress. The optimized current density for spalling a 50 μm silicon wafer was 5 mA/cm^2^. The stress induced in the silicon wafer, was calculated from the shift in the XRD peak. The main peak of the silicon wafer was shifted from 2θ of 68.88° to 69.28° by the stress of the silicon wafer, indicating that the silicon wafer was under compressive stress. However, the scale of the peak shift was too small to allow an accurate calculation of the stress in the silicon wafer (Figure 3C).

### Initial Crack Formed in Silicon Wafer by Laser Process

**Figure 4:**
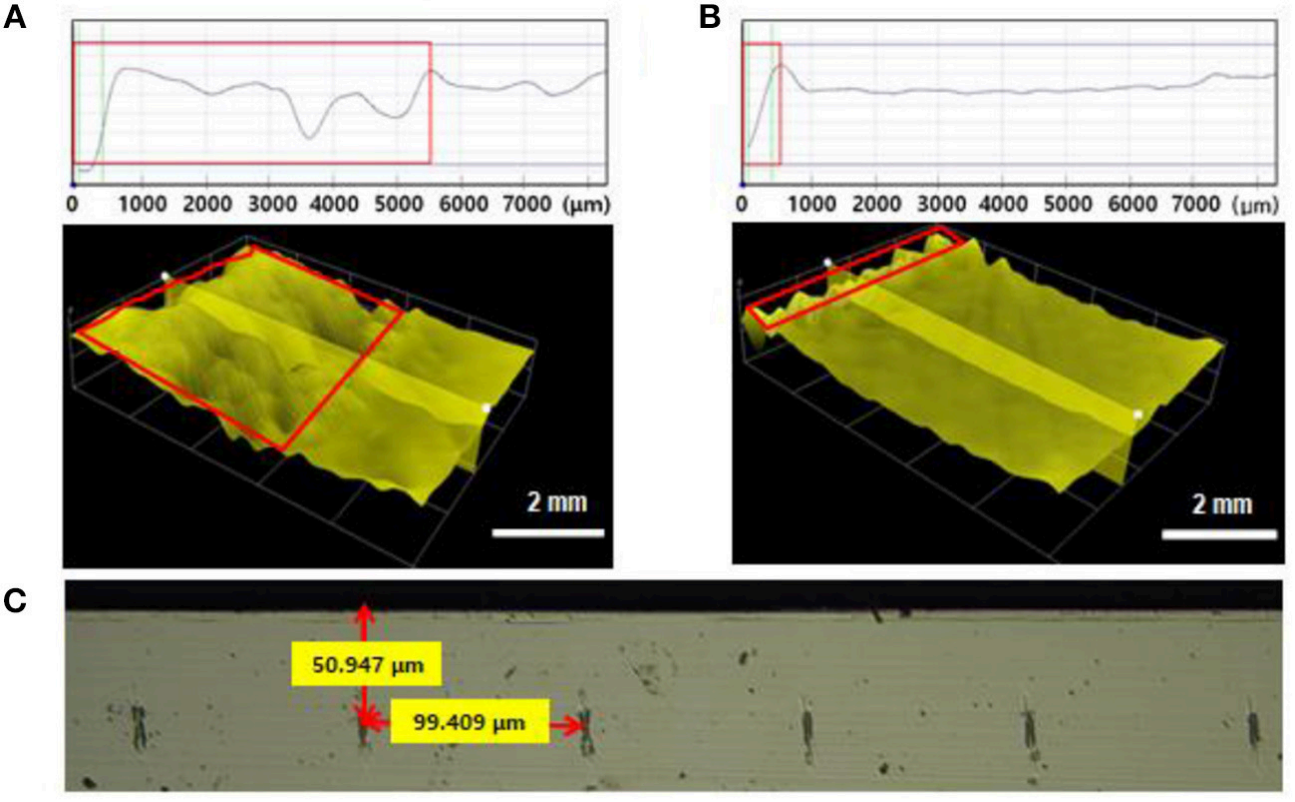
Surface profiling (top) and 3D optical microscope image (bottom) of the edge slope of spalled silicon wafers: **(A)** without laser pretreatment; **(B)** with laser pretreatment. **(C)** Image of initial cracks (50 μm).

Based on our findings, we suggest fabricating kerf-less silicon wafers via a laser process to decrease the kerf-loss area. Figure 4 shows the edge slope image of spalled silicon wafers prepared with and without laser pretreatment. The edge of the spalled silicon wafer without the laser pretreatment had non-uniform roughness and an edge-slope width of 5,500 μm. The edge-slope width of all samples prepared without the laser treatment had the same area, regardless of the sample size. On the other hand, the laser-treated spalled silicon wafer had an edge-slope width of 500 μm, as shown in Figure 4B. The area of the edge slope was <10% than that of the sample without the laser pretreatment because of the trajectory formed at the edge slope of the spalled silicon wafer. A spalled silicon wafer generally exhibits an unstable trajectory toward the fracture threshold before the steady-state crack depth. To reach the steady-state crack depth for a stable-state trajectory, crack propagation must be initiated at the interface between the silicon wafer and the nickel stressor layer. The depth of the initial cracks could be controlled by focusing on the edge, as shown in Figure 4C. By adjusting the laser focus, the initial cracks were produced at points that had the same depth and the steady-state crack depth. These initial cracks led to a stable-state trajectory, and crack propagation was initiated at the steady-state crack depth. The trajectory initiated from the silicon wafer edge by the laser process, was parallel to the interface between the silicon wafer and the nickel stressor film with biaxial tensile stress (Rice, 1988). Consequently, we confirmed that the initial crack formed by the laser process led to a decrease in the kerf-loss area of the silicon wafer.

### Spalled Kerf-Less Silicon Wafer

**Figure 5:**
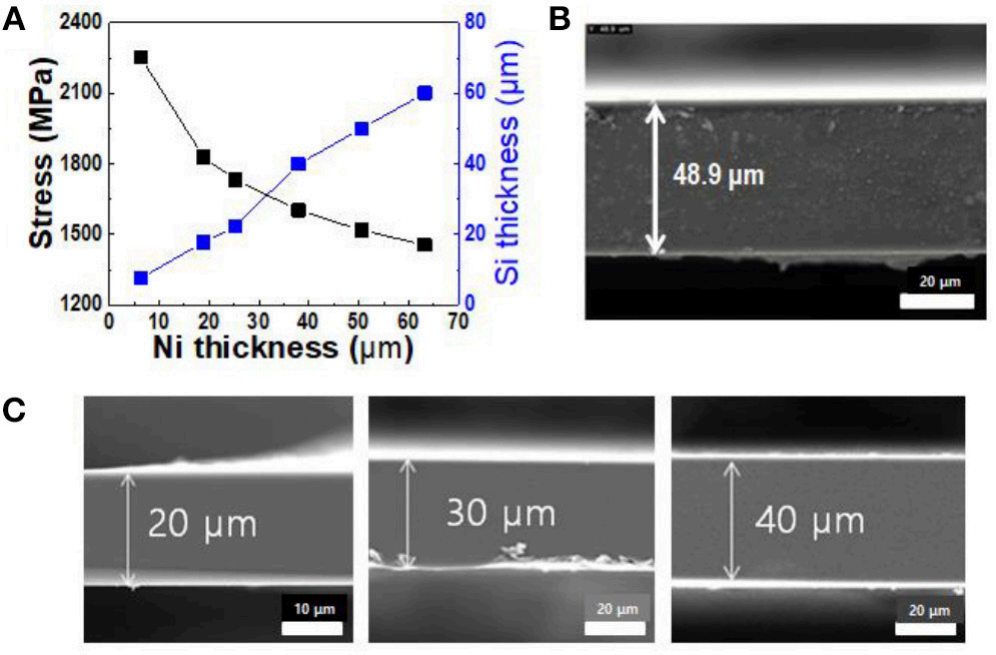
Thickness of spalled silicon wafers corresponding to nickel stressor layers of various thickness: **(A)** variations in thickness of spalled silicon wafer with changes in stress and nickel layer thickness; **(B)** SEM cross-sectional image of spalled silicon wafer (156 × 157 mm^2^); **(C)** spalled silicon wafers of various thickness.

Figure 5A shows the thickness of spalled thin silicon wafers and the induced stress as functions of the nickel thickness. The induced stress in the silicon wafer decreased with increasing nickel thickness, resulting in increased thickness of the spalled silicon wafer. Because the induced stress in the silicon wafer caused crack propagation inside the silicon wafer at a steady-state crack depth, the thickness of the spalled silicon wafer was higher when the internal stress of the nickel stressor layer was lower. As shown in Figures 5A,C, the thickness of the spalled silicon wafers was in the range of 20 to 70 μm. Once a crack was initiated in a silicon wafer, it could propagate in a direction parallel to the surface at a depth proportional to the thickness of the nickel layer at the center of the wafer. Figure 5B shows an SEM cross-sectional image of a spalled silicon wafer with a 50 μm nickel stressor layer. It should be noted that this nickel stressor layer was removed by wet etching, leaving a spalled silicon wafer with a flat surface and a thickness of 50 μm.

**Figure 6:**
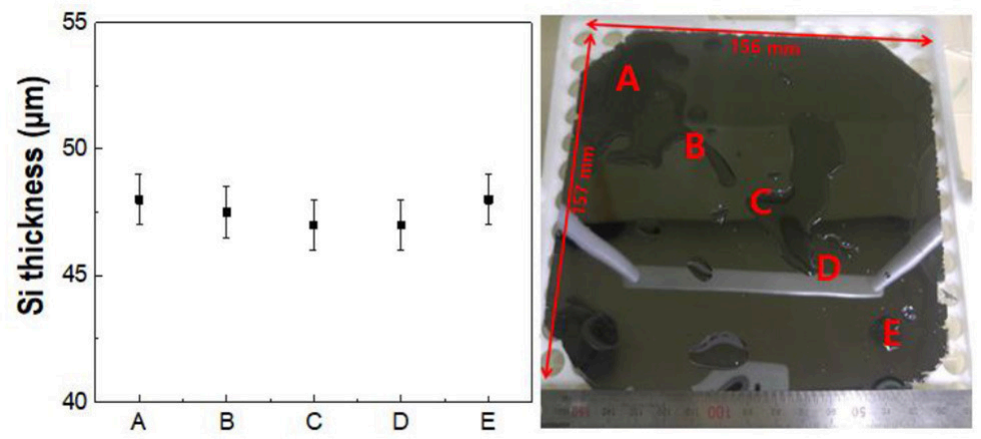
Thickness distribution on a 150 × 150 mm^2^ (6 × 6 in^2^) large-area spalled silicon wafer; image of a kerf-less ultra-thin silicon wafer.

To verify the thickness distribution of the spalled silicon wafer with a large area, the spalling process was carried out on a 150 × 150 mm^2^ (6 × 6 in^2^) silicon wafer. Figure 6 shows that a thin silicon wafer with a large area was obtained. The thickness was measured at five points on the silicon wafer, and each point on the same line was plotted at intervals of 220 mm. The spalled silicon wafer in Figure 6 exhibited a uniform thickness of approximately 50 μm, and the deviation of the silicon wafer thickness remained under 2 μm (4%). This uniform thickness resulted from the induced homogenous stress, and we believe that the approach can be used to provide significant performance improvements in large-scale silicon production.

**Figure 7:**
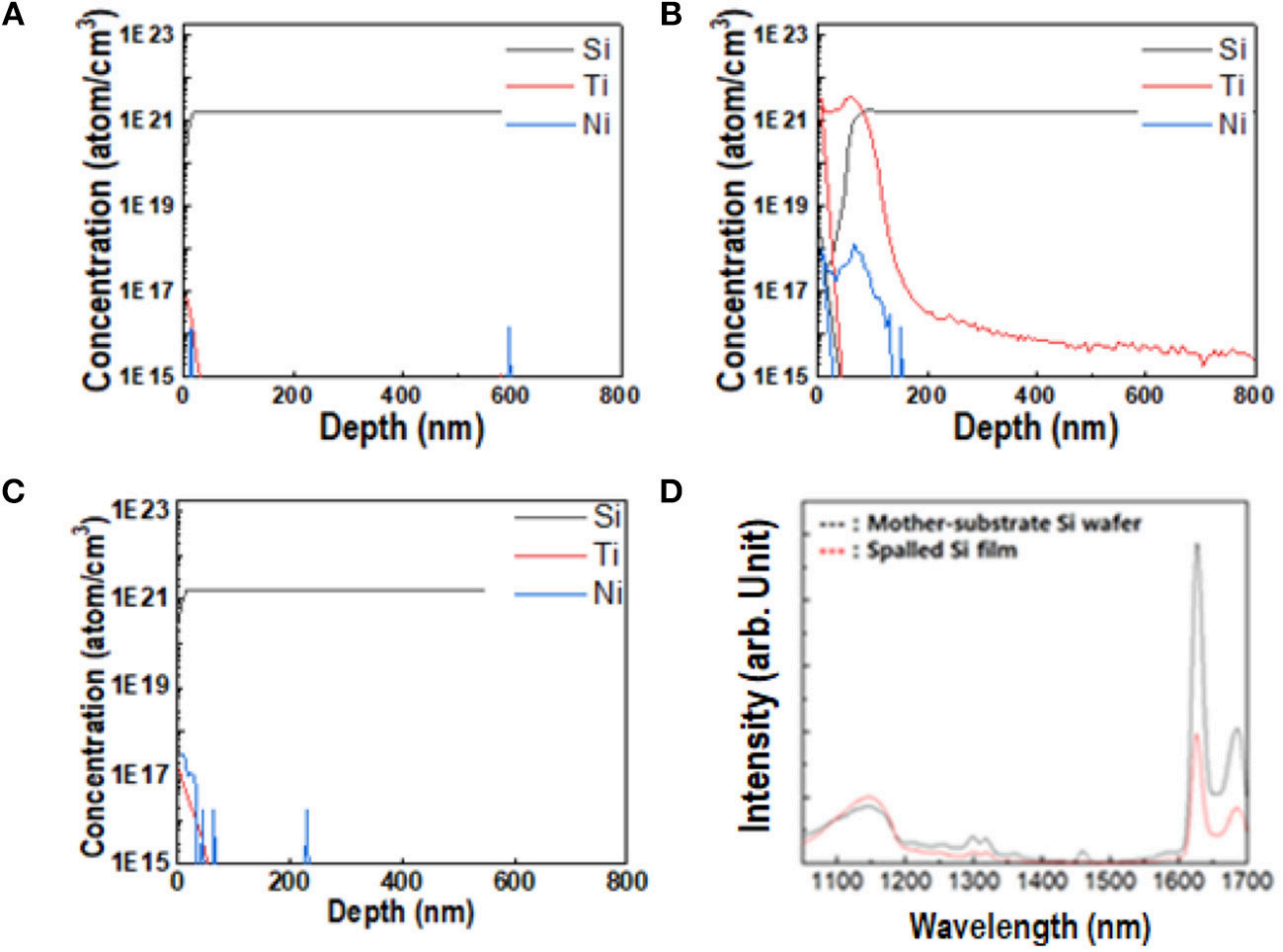
Physical properties of spalled Si wafer. **(A–C)** Atomic concentration of Si, Ti, and Ni at various vertical positions in different samples: **(A)** pure Si mother substrate; **(B)** spalled Si wafer after etching without HF; **(C)** spalled Si wafer after etching with 25 mL/L HF. **(D)** Comparison of PL of spalled Si wafer (red line) with that of pure Si mother substrate (black line).

For the successful application of spalled wafers in silicon-based solar cells, impurities that diffused into the silicon wafer during evaporation or electrodeposition, must be monitored as they could affect the mechanical and optical properties of the silicon, thus compromising the efficiency of the solar cell. Figure 7 shows the physical and optical properties of a spalled silicon wafer after each etching process. There were no impurities on the bare silicon wafer, as shown in Figure 7A. In general, the detection limit of most instruments is in the range of 10^13^-10^15^ atom/cm^3^^1^. As shown in Figure 7A, 10^17^ atoms of Ti were detected in 1 cm^3^ of the pure silicon wafer. Therefore, in this study, the detection limit of Ti and Ni atoms was assumed to be 10^17^ atom/cm^3^. After the nickel etching process without the HF solution, a Ti layer and a small Ni peak was detected on the spalled silicon wafer (Figure 7B). The Ni ions were expected to penetrate the Ti layer during the evaporation or electrodeposition. To eliminate Ni-based impurities, the Ti layer must be etched. Figure 7C shows no impurities on the spalled silicon wafer after it was etched with a HF solution. Figure 7D shows the PL spectra of both the pure silicon wafer and the spalled silicon wafer. The spalled silicon wafer showed no obvious PL spectrum shift, when compared to the spectrum of the pure silicon wafer, indicating that the band structure related to PL of the spalled silicon wafer remained unchanged by spalling.

### Analysis of Steady-State Crack Depth

According to the S&H model, the thickness of a spalled silicon wafer can be predicted when the initial crack can be calculated from the stress induced by the electrodeposited layer. There, internal stress of electrodeposited materials during the spalling process, can be traced to two origins. The first source is the misfit stress resulting from the lattice mismatch between the substrate and the metal film (Sun and Jih, 1987; Saitou, 2008). The induced misfit stress in the substrate tends to reduce the potential energy and causes its own curvature and fracture (Richardson, 2014). This phenomenon manifests as changes in the energy release rate (Kim et al., 1996), which is the energy dissipated per unit of a newly created fracture surface area (i.e., the tip) during a fracture^2^. A fracture causes free-state stress on the upper side, which is the opposite side of the mother substrate.

This energy release rate, *G*, is defined as

(1) $$\begin{array}{r} G=\frac{\partial \left(U-V\right)}{\partial A}, \end{array}$$

where *U* is the potential energy available for crack propagation, *V* is the work associated with any external forces acting on the system, and *A* is the crack area (linear for two-dimensional cracks). The crack direction is along the direction of the tip. When the fracture energy, *G*_c_, is higher than *G (G*_c_ > *G*), the crack begins to propagate. *G*_c_ is considered to be a material property that is independent of the applied load and the geometry of the body. In order to apply the stress needed for spalling, the external force must be zero, i.e., *V* = 0, which means that the crack propagation occurs on its own. If the external force is not zero, the spalled wafer will have high roughness.

**Figure 8:**
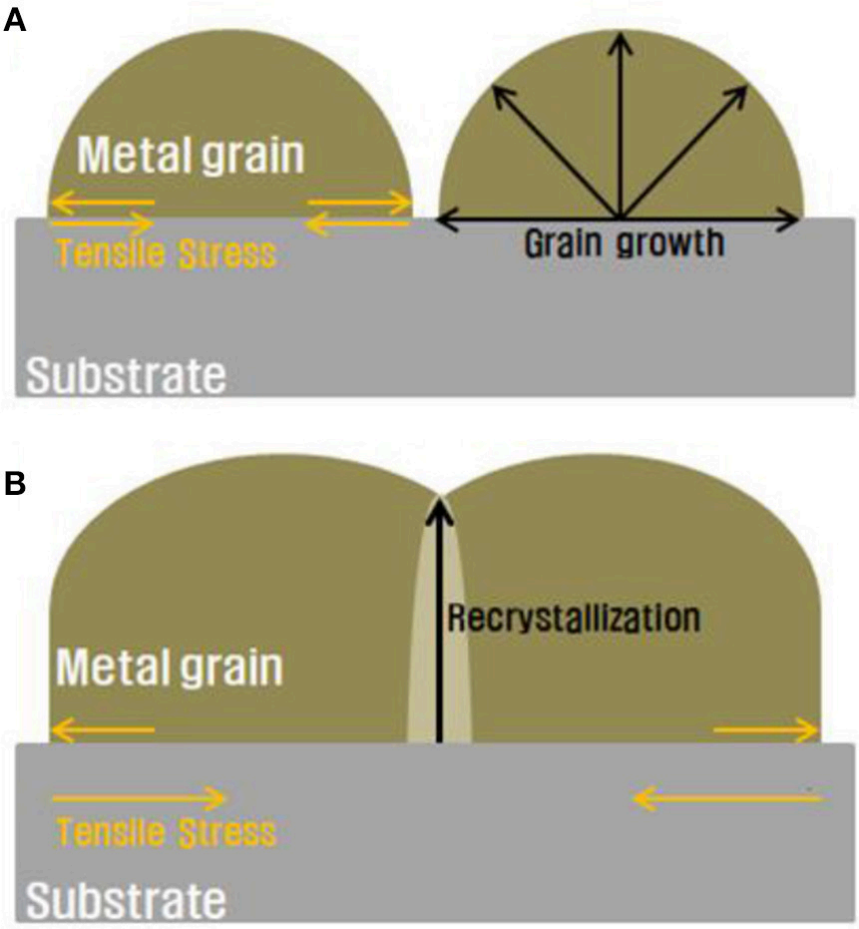
Schematic of residual stress induced by **(A)** isolated metal particles and **(B)** coalescence of metal particles on the substrate.

Internal stress can also be induced when coalescence occurs in a single material. The intrinsic stress of the coalescence of metal grains (He and Hutchinson, 1989) is caused by the nucleation of isolated states that grow and approach other grains. When a grain encounters other grains, recrystallization occurs at the grain boundary (Sun and Jih, 1987) This recrystallization area has a different crystal direction when compared with the original grains (Rachwal, 2010), resulting in stress in the metal film. To induce stress inside a silicon substrate in our study, an electrodeposited nickel layer was used as the stressor layer. At the initial stage, isolated nickel grains were formed on the silicon substrate and each grain grew at the same rate to form the nickel stressor layer. Because the nickel layer had a smaller lattice constant than that of the silicon substrate, tensile stress was induced in the silicon substrate Indenbom and Kaganer, 1990). Because the deposited nickel particles on the silicon substrate had different crystal growth directions, there was a lattice mismatch between the silicon wafer and every grain with a single-crystalline structure (Figure 8). Consequently, spalling stress was formed in the silicon wafer (Moridi et al., 2013). Because the stress of the nickel layer can be controlled as a function of the layer thickness, we expected to be able to predict the steady-state crack depth of the silicon wafer from the stress induced by the nickel layer.

**Figure 9:**
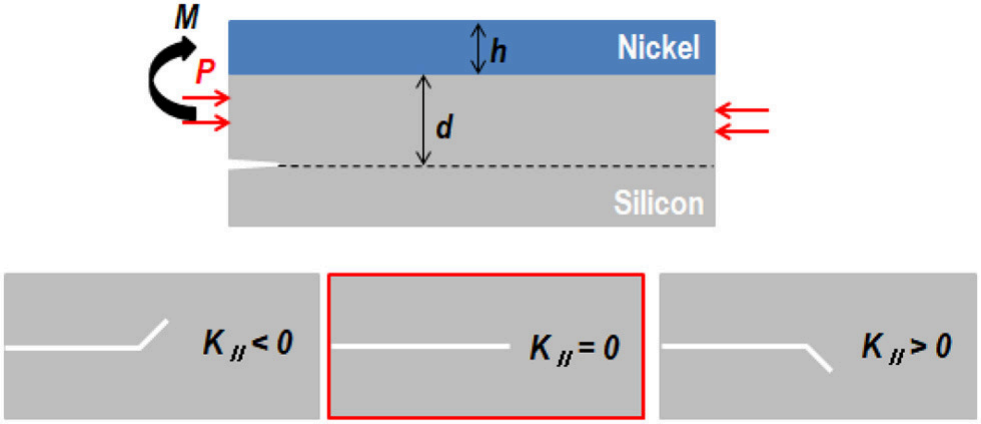
**(A)** Edge load and momentum of the Ni/Si thin-film system. **(B)** Trajectory of crack propagation in terms of *K*_II_ value.

To estimate the growth of the initial crack depth to the steady-state value, the steady-state crack depth must be calculated before the formation of the nickel stressor layer. Since the initial crack depth was determined by material properties such as Dundurs' elastic parameter and the stress intensity factor, it could be calculated using the Ni/Si thin-film system and any other material in the system. The calculation proposed here is based on the S&H model (Evans and Hutchinson, 1984; Suo and Hutchinson, 1990; DEAS Harvard University) and the kinking mechanism (Rice, 1988; Martini et al., 2012; Kwon et al., 2013). The starting point for calculating the steady-state depth is the energy-release rate, *G*, which is determined by the strain value of the beam (*P, M, d*) and the trajectory of propagation with stress intensity factors, *K*_I_ and *K*_II_:

(2) $$\begin{array}{r} G=\left[P^{2}+12{\left(\frac{M}{d}\right)}^{2}\right]/2Êd=\frac{\left(K_{I}^{2}+K_{II}^{2}\right)}{Ê}, \end{array}$$

where *d* and *h* are the spalled silicon wafer thickness and electrodeposited nickel thickness, respectively; *P* is the edge load; *M* is the momentum of the beam; and Ê is the strain or stress (Figure 9). The factor *K*_I_ is the minimum condition for the propagation of a crack caused by the nickel stressor layer. Otherwise, *K*_II_ expresses the direction of propagation. Since the spalling method is based on an existing crack, the *K*_I_ value is positive and will change with *K*_II_ values. Otherwise, if there are no external forces, the direction of crack propagation is parallel to the interface between the silicon wafer and the nickel stressor layer. For multilayered thin-film systems, individual stress intensity factors can be assigned to each film layer and solved separately.

(3) $$\begin{array}{rcl} K_{I} & = & c_{1}\frac{P}{\sqrt{d}}+c_{2}\frac{M}{\sqrt{d^{3}}}, \end{array}$$

(4) $$\begin{array}{rcl} K_{II} & = & c_{3}\frac{P}{\sqrt{d}}+c_{4}\frac{M}{\sqrt{d^{3}}}, \end{array}$$

where *c*_1_*, c*_2_*, c*_3_, and *c*_4_ are dimensionless constants. These constants can be calculated using the loading conditions as follows:

(5) $$\begin{array}{r} c_{1}^{2}+c_{3}^{2}=\frac{1}{2}, \end{array}$$

(6) $$\begin{array}{r} c_{1}c_{2}+c_{3}c_{4}=0, \end{array}$$

(7) $$\begin{array}{r} c_{2}^{2}+c_{4}^{2}=6. \end{array}$$

To satisfy these loading conditions, *c*_1_ = 0.434, *c*_2_ = 1.934, *c*_3_ = 0.558, and *c*_4_ = −1.503 were used (Evans and Hutchinson, 1984), with *c*_4_ being a negative value. Because *K*_II_ is a complex number, *d* can be obtained from

(8) $$\begin{array}{r} K_{I}=0.434\frac{P}{\sqrt{d}}+1.934\frac{M}{\sqrt{d^{3}}}, \end{array}$$

(9) $$\begin{array}{r} K_{II}=0.558\frac{P}{\sqrt{d}}-1.503\frac{M}{\sqrt{d^{3}}}. \end{array}$$

Because the trajectory of the tip in a spalling process is parallel to the interface between the nickel layer and the silicon wafer, *K*_II_ must be zero. Therefore, the *P*/*M* ratio has the same shape as the function for *d*.

The *h* formation can be represented as

(10) $$\begin{array}{r} K_{I}=\frac{P}{\sqrt{2h}}\text{cos}w+\frac{2\sqrt{3}M}{\sqrt{2h^{3}}}\text{sin}w, \end{array}$$

(11) $$\begin{array}{r} K_{II}=\frac{P}{\sqrt{2h}}\text{sin}w-\frac{2\sqrt{3}M}{\sqrt{2h^{3}}}\text{cos}w, \end{array}$$

where *w* is the mode mixity for a complex number of the stress intensity factor (Suo and Hutchinson, 1990). To obtain the value of *w*, Dundurs' elastic parameter (α) is needed. It can be calculated as follows (Suo and Hutchinson, 1990):

(12) $$\begin{array}{r} \alpha =\frac{\text{Γ}\left(\kappa_{2}+1\right)-\left(\kappa_{1}+1\right)}{\text{Γ}\left(\kappa_{2}+1\right)+\left(\kappa_{1}+1\right)}, \end{array}$$

where *Γ* is the shear modulus ratio, κ_1_ = 3 – 4*v*_1_, κ_2_ = 3 – 4*v*_2_, *v*_1_ is Poisson's ratio of nickel, and *v*_2_ is Poisson's ratio of silicon. Dundurs' elastic parameter has various values ranging from 0 to 1, and it only depends on the intrinsic properties of the material. This theoretical approach can be applied to various materials. For a silicon wafer with a nickel stressor layer, *w* is 52 when α is 0.4 (obtained from the mode mixity table of the S&H model) (Suo, 1989). The term *h* can also be represented as *P/M* = *g*(*h*) when *K*_II_ = 0. As a result, we have the equation *d* = λ*h*, where λ is a proportional factor. For the Ni/Si thin-film system, λ = 0.99, which means that the thickness of the spalled silicon layer was proportional to that of the nickel layer. This proportional relationship originates from the standard properties (from modulus ratio to elastic parameter) of nickel. Moreover, *h* can be expressed in terms of stress. Therefore, the thickness of a spalled silicon wafer can be calculated regardless of the unstable factors. *d* is expressed in terms of stress as

(13) $$\begin{array}{r} d=\frac{\mu \lambda }{S}U_{\left(h\right)}, \end{array}$$

where *μ* is a constant originating from the ratio between the modulus of the substrate and that of the film, *U*_(h)_ is the curvature of the beam, and *S* is the intrinsic stress. Furthermore, *U*_(h)_ can be calculated using the equation

(14) $$\begin{array}{r} {\text{U}}_{\left(\text{h}\right)}=C\text{urvature rate}\times {\left(\frac{h-h_{\text{e}}}{\text{Deposition rate}}\right)}^{\text{Degradation of curvature}}, \end{array}$$

where *h*_e_ is the initial thickness of the nickel layer (adhesion layer). In this equation, the curvature rate shows the amount of the upper beam that has been curved, while the degradation of curvature shows changes in the curvature rate as a function of the thickness of the nickel stressor layer. As a result, a new equation can be obtained for predicting the thickness of a spalled silicon wafer. To confirm the reliability of the new equation, the calculated value was carefully compared with the experimental result.

**Figure 10:**
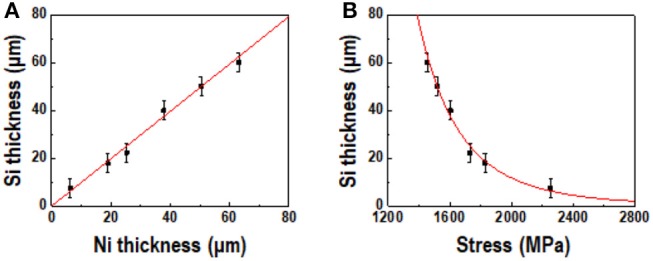
Comparison of theoretical (red line) and experimental (black dots) values of silicon thickness as functions of **(A)** Ni layer thickness and **(B)** stress induced by Ni layer.

Figure 10 shows a comparison of the theoretical and experimental values of thickness of the spalled silicon wafer as functions of the nickel layer thickness and stress. The red line shows the fitted line based on the calculated value, while the black dots represent the experimental results. As shown in Figure 10A, the thickness of the silicon layer increased proportionately with the thickness of the deposited nickel layer. A calculated value of λ = 0.99 was obtained from the red line, which nearly coincides with the experimental value. Figure 10B shows the calculated and experimental values of the silicon wafer thickness as functions of the stress induced by the intrinsic stress in the nickel layer. The results show that the thickness of the spalled silicon wafer was inversely proportional to the stress, which means that the induced stress in the silicon wafer increased with decreasing internal stress in the nickel layer. The discrepancy between the calculated and experimental results is under 1.1%; this low error may have originated from the roughness of the nickel layer and *K*_II_ not being a perfect zero during the spalling process because of external forces. Kerf-less silicon wafers of the desired thickness were successfully fabricated from the calculated value of the steady-state crack depth and initial crack depth predicted by the new equation, providing a noticeable enhancement in the performance of the kerf-less silicon device.

### Fabrication of Solar Cells Using the Spalled Si Wafer

**Figure 11:**
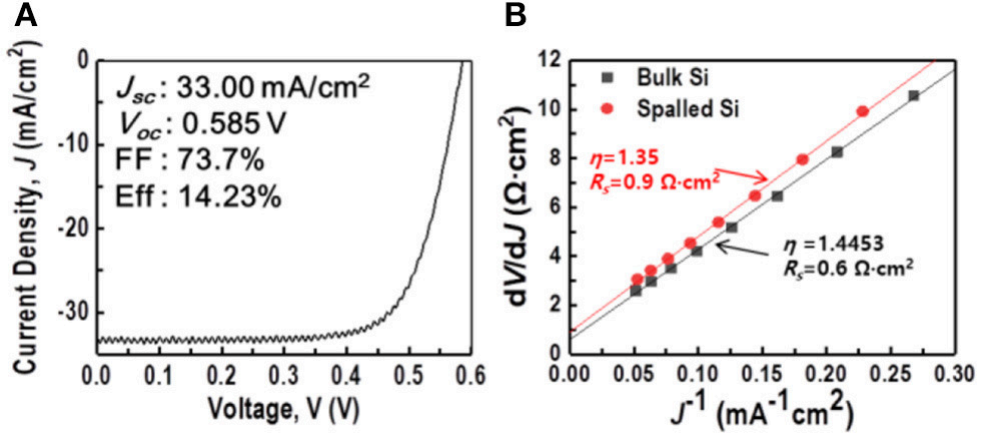
*J–V* characteristics of spalled-silicon solar cell: **(A)** standard *J–V* curve; **(B)** *r*(*J*) with fitted results used to determine *R* and *A* at room temperature.

**Figure 12:**
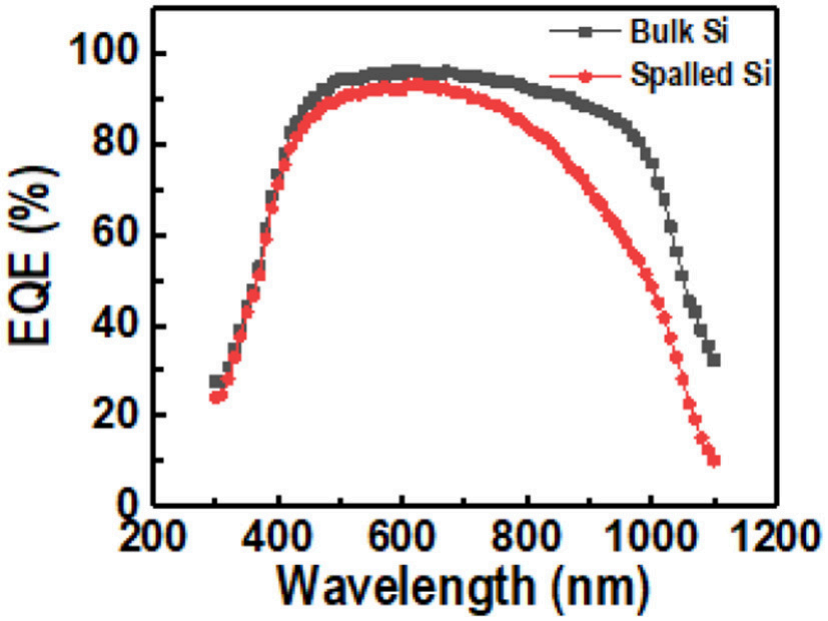
External quantum efficiency (EQE) of both spalled silicon wafer and bulk silicon wafer.

Several parameters such as the ideality factor (η), quantum efficiency (EQE), and conversion efficiency (Eff) were evaluated to determine the applicability of the spalled silicon wafer. The results are shown in Figures 11, 12.

As shown in Figure 11A, the efficiency of the resulting solar cell was 14.23%. The short-circuit current density, *J*_sc_, which was determined by the light absorption and quantum efficiency, was 33 mA/cm^2^. It is well known that the performance of a silicon-based solar cell is affected by defects originating from impurities in the materials. These impurities can lead to shallower- and deeper-energy defect levels in the band structure, which can create recombination paths that are different from band-to-band recombination. This means that if there were defects on the spalled silicon wafer, degradation of performance could be measured from the defects in the band structure. In order to check the degradation of performance, the ideality factor of the spalled silicon wafer was compared with that of a bulk silicon wafer (obtained from the Shockley diode equation Hegedus and Shafarman, 2004 and the general single-exponential diode equation), as shown in Figure 11B. The plot of the derivative d*V/*d*J* vs. *J*^−1^ was obtained from the dark *J–V* curve to identify differences in the device characteristics, ideality factor η, and the series resistance *R*_*S*_ between the solar cells, fabricated utilizing a bulk Si wafer and a spalled Si wafer according to the following equation (Hegedus and Shafarman, 2004):

(15) $$\begin{array}{r} J=J_{0}\text{exp}\left[\frac{q}{\eta kT}\left(V-R_{s}J\right)\right]+G^{\prime }V, \end{array}$$

where *J*_0_ is the dark saturation current, *q* is the electrical charge, η is the ideality factor, *k* is the Boltzmann constant, *T* is the temperature in Kelvin, *R*_S_ is the series resistance, and *G'* is the shunt conductance. Since *G'* is supposed to be negligible, the derivative plot could be extracted through a simplified equation without *G'* as follows (Hegedus and Shafarman, 2004):

(16) $$\begin{array}{r} r\left(J\right)\equiv \frac{dV}{dJ}=R_{s}+\frac{\eta kT}{q}J^{-1}. \end{array}$$

Equation 16 was plotted as a linear line with a slope of ηkT/q and a *y*-intercept of R_S_. η was calculated using the slope and a thermal voltage (*kT/q*) of 25.69 mV at room temperature. As shown in the comparison of device characteristics Figure 11B of the solar cells using a bulk silicon wafer and a spalled silicon wafer, the measured values of *R*_*S*_ were 0.6 and 0.9 *Ω*·cm^2^, respectively, and the measured values of η were 1.45 and 1.35, respectively. Even though the same fabrication processes were used, the calculated results of *R*_*S*_ and η for the solar cell using a spalled wafer, were higher than those for the solar cell using a bulk wafer. This increase was attributed to the wafer thickness because the thinner the wafer, the larger the effect of rear-side recombination. The ideality factor η of the solar cell using a spalled silicon wafer was 1.35, which is closer to 1 when compared with η of the solar cell using a bulk silicon wafer. This means that the spalling process for preparing thin silicon solar cells did not form cracks and defects in the band structure. It also appears that the lower ideality factor was due to the shunt resistance during the fabrication of the solar cell. Moreover, the EQE of the cells using a spalled silicon wafer and a bulk silicon wafer (mother substrate) was measured; the results are shown in Figure 12. The absorption of light in the wavelength range of 400–550 nm was the same for both cells. In the wavelength range above 550 nm, the difference in light absorption by both cells was more distinct, because the light-absorption efficiency was related to the silicon thickness (Green and Keevers, 1995). The lower EQE was also affected by the rear-side recombination in the spalled silicon film, which had a thickness of 50 μm.

## Conclusions

A kerf-less thin silicon wafer with a large area was successfully fabricated by spalling, and its thickness was calculated from the steady-state crack depth, using the proposed equation based on the Suo–Hutchinson model and the kinking mechanism. A nickel stressor layer was uniformly deposited on the silicon wafer with the assistance of a shield. It displayed a uniform thickness of about 7 μm and exhibited excellent electric properties. In order to create the initial crack for decreasing kerf-loss in a large-area thin silicon, the silicon wafer was pretreated with a laser before the spalling process. We confirmed that the kerf-loss area of a laser-treated silicon wafer, was < 10% of the kerf-loss area of a spalled silicon wafer without pretreatment. The thickness of the spalled silicon wafer varied from 20 to 70 μm. The silicon layer thickness increased proportionately with the nickel layer thickness, while it was inversely proportional to the stress. The predicted thickness calculated using the proposed equation is in agreement with the experimental value. Finally, the solar cell fabricated with a spalled silicon wafer had an efficiency of 14.23% and an ideality factor of 1.35.

## Author Contributions

All authors assisted in the development and writing of the paper. In addition, H-SY, JK, SeK, NE, SP, BY, and J-HL were involved in designing and doing the experiment. C-SH, SHK, and DL did laser process at the edge of silicon wafer. SaK and J-HL made the solar cell using the exfoliated silicon wafer and measured the solar cell efficiency. JC and C-LL measured and analyzed the optical properties of the exfoliated silicon wafer.

### Conflict of Interest Statement

The authors declare that the research was conducted in the absence of any commercial or financial relationships that could be construed as a potential conflict of interest.

## Footnotes

^1^Evans Analytical Group. Available online at: http://www.nanoscience.co.jp/industry_analysis/pdf/AN339.pdf

^2^DEAS Harvard University. Available online at: http://www.mrsec.harvard.Edu/education/ap298r2004/Vlassak%20AP298presentation.pdf

## Abbreviations

ALD: atomic layer deposition
DI: deionized
IPA: isopropyl alcohol
ITO: indium–tin oxide
PL: photoluminescence
S&H: Suo and Hutchinson
SEM: scanning electron microscopy
SIMS: secondary ion mass spectroscopy
XRD: X-ray diffraction.
