# Supplementary material for: Kerf-Less Exfoliated Thin Silicon Wafer Prepared by Nickel Electrodeposition for Solar Cells
Source: Front Chem. 2019 Jan 14;6:600. doi: 10.3389/fchem.2018.00600 (PMC6339913; doi:10.3389/fchem.2018.00600)
Supplement: Supplementary file 1 [file Table_1.DOCX]

Supplementary Material

**Kerf-Less Exfoliated Thin Silicon Wafer Prepared by Nickel Electrodeposition for Solar Cells**

**Hyun-Seock Yang^1,2^, Jiwon Kim^1^, Seil Kim^1^, Nu Si A Eom^1^, Sangmuk Kang^3^, Chang-Soon Han^4^, Sung Hae Kim^5^, Donggun Lim^3^, Jung-Ho Lee^5^, Sung Heum Park^2^,** **Jin Woo Choi^6^, Chang-Lyoul Lee^6^, Bongyoung Yoo^5*^, Jae-Hong Lim^1*^**

^1^Electrochemistry Department, Korea Institute of Materials Science, 797 Changwondaero, Changwon, Gyeongnam 51508, Republic of Korea.

^2^Department of Physics, Pukyong National University, Busan 608-737, Republic of Korea

^3^Department of IT Convergence, Korea National University of Transportation, 50 Daehak-ro, Chungju, Chungbuk 380702, Republic of Korea

^4^Laser Advanced System Industrialization Center, Samtae-ro, Mam-myeun, Jangseong-gun, Jeollanam-do, Republic of Korea

^5^Department of Materials Engineering, Hanyang University, Ansan 425-791, Republic of Korea.

^6^Advanced Photonics Research Institute (APRI), Gwangju Institute of Science and Technology (GIST), Gwangju 61005, Republic of Korea.

***Correspondence:**

E-mail: [lim@kims.re.kr](mailto:lim@kims.re.kr) (J.-H. Lim), [byyoo@hanyang.ac.kr](mailto:byyoo@hanyang.ac.kr) (B. Yoo).


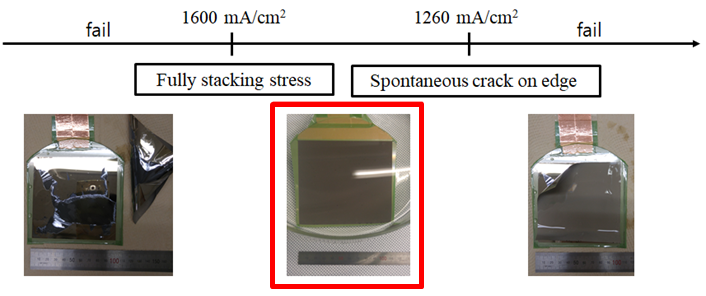


**Supplementary Figure 1.** Internal stress conditions for the stable spalling method.
